# The feasibility and acceptability of a primary school-based programme targeting diet and physical activity: the PhunkyFoods Programme

**DOI:** 10.1186/s40814-019-0542-2

**Published:** 2019-12-20

**Authors:** Pinki Sahota, Meaghan Christian, Rhiannon Day, Kim Cocks

**Affiliations:** 1 School of Clinical and Applied Sciences, Leeds Beckett University, Calverley Street, Leeds, LS1 3HE UK; 2 KCStats Consultancy, Leeds, UK

**Keywords:** Schoolchildren, Diet, Physical activity, Intervention, Childhood obesity

## Abstract

**Background:**

This study aims to evaluate the feasibility and acceptability of the PhunkyFoods Programme, a primary school-based intervention to promote healthy nutrition and physical activity knowledge and behaviours to assess outcomes to inform a phase 3 trial.

**Methods:**

The cluster randomised feasibility trial recruited eight primary schools from the North of England. Elibility criteria included all primary schools in one town, excluding independent and special schools and schools that comprised of only key stage 2 pupils (years 3–6). Eight schools agreed to participate. Randomisation to intervention or control arms was in a 1:1 ratio. Intervention schools received PhunkyFoods over 17 months. Control schools continued with usual curriculum. Assessors were blinded to group assignment. Measures comprised of a Healthy Lifestyle Knowledge Questionnaire and Synchronised Nutrition and Activity Program to assess diet and physical activity, height, weight, and psychological wellbeing. Feasibility outcomes were recruitment, attrition rates, interviews with teaching staff, focus groups with pupils to explore the acceptability of outcome measures, implementation, intervention content, and programme fidelity.

**Results:**

Three hundred fifty-eight pupils, aged 6–9 years from eight schools were recruited at baseline (control *n* = 170, intervention *n* = 188); 337 (94.1%) at 6 months (control *n* = 163, intervention *n* = 181); and 331 (92.5%) at 18 months (control *n* = 152, intervention *n* = 179), and 6 pupils opted out. Trends in increased knowledge of healthy lifestyle behaviours, healthier eating, and liking of fruit and vegetables were reported in the intervention compared to the control group. Year 4 intervention pupils had significantly higher healthy balanced diet knowledge scores compared to control pupils, mean difference 5.1 (95% CI 0.1 to 10.1, p=0.05). At 18 months, the mean percentage of vegetables liked was higher (intervention 53.9% vs. 43.0% control). Similarly, percentage of fruits liked was also higher (intervention 76.9% vs. 67.2% control). Qualitative data showed that delivery of the intervention was feasible and acceptable to teachers and pupils. Lessons were learned to inform the phase 3 trial around the dietary assessment measure and timing of recruitment.

**Conclusions:**

Whilst the study was not powered to detect a definitive effect, results suggest a potential to increase knowledge of healthy lifestyle behaviours and dietary behaviours, suggesting that with minor changes, a phase 3 trial is likely to be deliverable.

**Trial registration:**

ISRCTN, ISRCTN15641330. Registered 8 May 2015—retrospectively registered, 10.1186/ISRCTN15641330

## Background

The impact of unhealthy lifestyles due to poor nutrition and sedentary behaviour in children is a major public health issue across the globe and of particular concern is the rise in childhood obesity [1, 2]. In England, 22% of 4–5-year-olds starting school are overweight and obese rising to 33% by the end of primary school (10–11 years) [3]. Childhood obesity impacts adversely on health, which continues into adulthood [4, 5]. An obese child is more likely to become an obese adult [6], and childhood and adolescent obesity is linked to ill-health and early death [6]. Obese children are more likely to experience psychological comorbidities such as depression and poorer perceived lower scores on health-related quality of life, emotional and behavioural disorders, and self-esteem during childhood compared to non-obese children [7]. The risk of psychological morbidity increases with age [8]. Obesity is difficult to reverse [9] thus strengthening the case for primary prevention. TV viewing/screen-based activity [10]; low levels of physical activity [11]; and high consumption of dietary fat, carbohydrates, and sweetened fizzy drinks [12] have been identified as common and modifiable risk factors that can be easily targeted in school-based interventions.

Schools are recognised as an ideal setting to address obesity and the associated risk factors given their ability to reach nearly all children who spend a significant proportion of their time in schools [13]. Moreover, the school environment offers physical facilities (e.g. playgrounds, dining rooms, food provision) and the opportunity for young children to be taught through experiential learning in order to establish healthy lifestyle behaviours, potentially leading to improved health outcomes in childhood and later in life.

Several systematic reviews [14–17] have identified school-based interventions aimed at 6–12-years-olds are effective at reducing adiposity. School-based interventions that involve physical activity as an essential component along with nutrition education may be effective in reducing children’s body mass index [2, 17]. Educational based interventions, which tended to focus more on diet and health knowledge outcomes, have showed statistically significant improvement in behaviours and knowledge of healthy eating, nutrition, and physical activity amongst children [18]. Another systematic review reported that experiential learning strategies were associated with the largest effects in reducing food consumption or energy intake, increasing fruit and vegetable consumption or preference, and increasing nutritional knowledge outcomes [19].

It is evident that providing school-based nutrition and physical activity interventions can lead to the formation of healthier eating habits and increased physical activity at school and at home. Successful intervention studies tended to involve training for teachers and staff delivering the intervention, integrating the intervention components into the school curriculum, as well as parental involvement through homework activities, and developing a whole school approach through adjustments to school policy around nutrition and physical activity education [18, 20]. Two of the key components to a successful intervention are the schools’ head teachers’ perspectives on the importance of healthy eating and whether it can be made a priority across the school [21, 22].

Despite the above evidence, few school-based interventions have been conducted in the United Kingdom (UK), which limits their generalizability to the UK primary school education system. As no single intervention will fit all school populations, further research needs to identify programmes including specific programme characteristics predictive of success across different contexts and countries. Additionally, information is required on intervention effects on the mediators of obesity (diet, physical activity, sedentary behaviour, and knowledge) within school settings [17]. The aim of this study is to evaluate the acceptability and feasibility of a school-based healthy eating and physical activity intervention (PhunkyFoods) in a primary school setting in the UK to promote knowledge and behaviours in healthy nutrition and physical activity. It also aims to ascertain the appropriateness of the measurements required to assess the outcomes in order to inform a phase 3 trial.

## Materials and methods

### Sample and recruitment

The sample size of eight schools (four intervention and four control arm) was based on the minimum recommended for a pilot cluster randomised trial [18]. A list of all primary schools within a town in the north of England was obtained from the Public Health Department. Independent and special schools and schools that comprised of only key stage 2 pupils (years 3–6; age 9–11 years) were excluded because of the likelihood of variations in curriculum delivery. All remaining eligible primary schools were invited and eight agreed to participate. All pupils in year 2 (aged 6–7 years) and year 4 (aged 8–9 years) at each school were invited to participate, to give a sample consisting of both key stage 1 (year 2) and key stage 2 (year 4) pupils, who would participate over two academic school years. Parents of pupils in year 2 and year 4 received a letter (opt-out consent) with information about the study 2 weeks prior to baseline data collection. Opt-out consent is the recruitment method successfully employed by the UK National Child Measurement Programme (NCMP) [20]. Parents who did not wish their child to participate completed the reply slip, which was then returned to the schools. Written consent for participation and audio-recording of the interviews was obtained from the head teachers, teachers, and catering staff. Pupil consent to audio-record the focus groups was also obtained before each session. Ethical approval was obtained through Leeds Beckett University Faculty Ethics Committee (Research Ethics Application number 283).

### Randomisation

Randomisation was carried out by a senior statistician at York Trials Unit, University of York. A minimisation algorithm was used to allocate schools to the intervention or control arm in a 1:1 ratio. Class size, social economic status (SES) (using free school meal (FSM) index as a proxy measure of SES), and ethnicity (using Black and Minority Ethnic (BME) status) were used as minimisation factors to balance the groups as well as possible given the small sample size. Mean class size, percentage BME, and percentage FSM were calculated from the identified schools and used as the cut-offs for the minimisation (Table 1).

**Table 1:** Balance across minimisation factors

Group	Class size		BME		FSM	
	< 25	≥ 25	≤ 25%	> 25%	≤ 17%	> 17%
Control	2	2	3	1	1	3
Intervention	2	2	3	1	3	1
Total	4	4	6	2	4	4

*BME* Black and Minority Ethnic, *FSM* free school meal eligibility (proxy for socio-economic status)

### Intervention

**Fig. 1:**
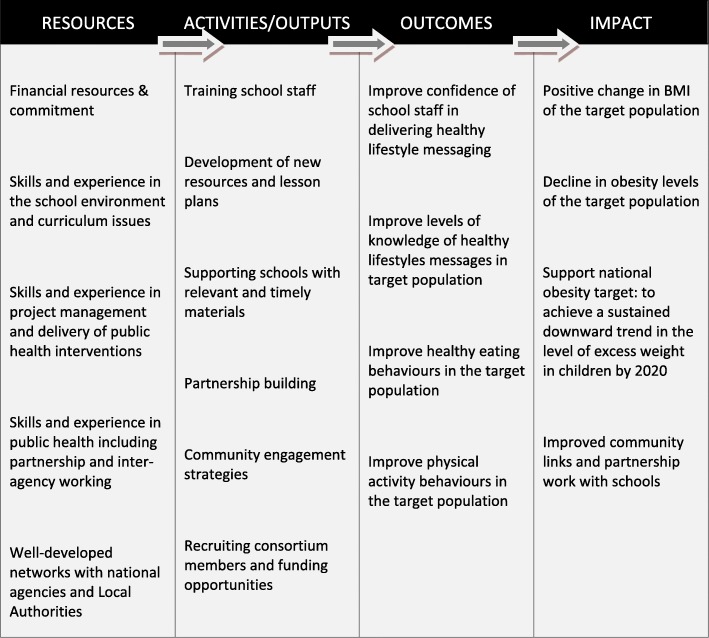
Logic model for the PhunkyFoods Programme

The programme ran for 17 months over 2 academic school years with the intervention delivered between February 2013 and July 2014 in the four schools randomised to receiving the intervention. The PhunkyFoods Programme (PFP) is evidence informed to target risk factors (diet and physical activity) aimed at the development of obesity and supports the whole school approach [23] to promoting health. It is underpinned by behavioural theory, mapped against the Behaviour Change Wheel (BCW) [23] and is comprised of intervention functions that impact on all three of the essential conditions for behaviour change, capability, opportunity, and motivation. The logic model is described in Fig. 1. The programme development has a strong patient public involvement of teaching staff and parents. The programme components include:

1. Capacity building by training school staff in healthy lifestyles teaching and delivery of the PFP for pupils and their families.
2. A flexible approach to delivery: designed to be delivered within the classroom or as a club, e.g. breakfast, after-school, or lunch club.
3. Designed to be delivered as weekly teaching sessions through embedding into the existing curriculum.
4. A wide selection of on-line, interactive cross-curricular healthy eating and physical activity lesson plans and a resource box comprising food models, food mats, food cards, DVDs, and books to facilitate teaching staff in programme delivery.
5. Increased sessions for physical activity and the development of fundamental movement skills throughout the school week.
6. The regional Community Support Consultants (CSC) offer ongoing support for teachers in the implementation of the whole school approach to healthy lifestyles.
7. Establishing environments and cultural practices that support eating healthier foods and being active throughout each day.
8. Parent support and home activities that encourage pupils to be more active, eat more nutritious foods, and spend less time in screen-based activities.

### Control schools

The control schools continued to deliver their existing curriculum and were offered £200 book vouchers (half at the end of year 1 and half at the end of year 2) as an incentive for their participation, as well as priority status to receive the PFP at the end of the study when the programme was to be offered to all primary schools in the area.

### Data collection

The following trial measures were recorded to inform the phase 3 trial: recruitment rate, barriers to recruitment, acceptability of randomisation, retention rates for schools and pupils across intervention and control groups, and reasons for dropouts. The feasibility of outcome measures was also assessed.

### Process measures

- Interviews (31 at 6 months; 29 at 18 months) were conducted in both intervention and control schools with head teachers, teachers, healthy school co-ordinators, catering staff, and one Community Support Consultant (who oversaw implementation of the PFP), to explore the following: acceptability of the PFP intervention, capability and capacity of schools to deliver and incorporate the intervention within the curriculum, and programme fidelity and sustainability.
- Sixteen focus groups (total 64 pupils; 32 intervention and 32 control) were conducted at 18 months (end of intervention) with the aim to evaluate pupil awareness of the programme, acceptability and impact of the PFP on pupils’ knowledge, and attitudes towards healthy eating and exercise. Each focus group was single sex and comprised of four mixed ability pupils nominated by the teacher. Two researchers conducted the focus groups during normal lesson time in a separate classroom for approximately 20–40 min using a standardised focus group topic schedule and stimulus food photographs (display of typical food for breakfast, snacks, packed lunch, and evening meals).
- A resources checklist co-designed with the programme provider, which listed all the available lesson plans from the PhunkyFoods curriculum, was used to evaluate the use and acceptability of the PFP lesson plans by the teaching staff. Year 2 and year 4 teaching staff were emailed the checklists to capture PFP implementation at 6 months follow-up. Additional paper copies of the resource’s checklists were handed out to year 3 and year 5 teaching staff during data collection at 18 months follow-up up as the children had changed year groups. Staff rated the acceptability on a 5-point Likert-type scale (1 = very poor, 2 = poor, 3 = acceptable, 4 = good, 5 = excellent).

### Baseline and follow-up measures

- Data was collected at baseline (pre-intervention), 6 months and 18 months (end of intervention). Age, sex, and ethnicity (from parent report at school entry) were obtained at baseline for all participating pupils.
- Healthy Lifestyle Knowledge Questionnaire (HLKQ) was a newly developed tool by the research team. The questionnaire was designed to evaluate pupils overall healthy nutrition and physical activity knowledge, which comprised of the following domains: nutrition knowledge, healthy/balanced diet knowledge, and physical activity knowledge. Together, these three domains created an overall healthy lifestyle knowledge score to measure health-related knowledge of participating pupils [19]. A validation study was conducted prior to the feasibility trial to assess validity and reliability of the HKLQ in 137 pupils with a mean age of 9 years (SD 1.3). The test-retest reliability was found to be good, with no statistically significant differences between time 1 and time 2 for any of the domains. The proposed domains had good internal consistency (Cronbach’s alpha 0.67 to 0.80) with the exception of the physical activity domain in years 4–5 (0.41) [unpublished results Christian et al.]. The questionnaires for both year groups were the same except that the year 4 questionnaire contained additional sections exploring pupils’ attitudes towards fruit and vegetables and physical activity. The questions on attitudes towards fruit and vegetables were developed using the Healthy Food Knowledge Activity Questionnaire [21], which has been validated in this age group [22]. The administration of the HLKQ also differed between year groups. For the year 2 pupils, the questionnaire was read out in class, to make sure every child understood each question, with pupils encouraged to ask for assistance if they were unsure of a particular question. The year 4 pupils completed the questionnaire independently in class but again were encouraged to ask for assistance if they were unsure of a question.
- Diet and lifestyle behaviour: the “Synchronised Nutrition and Activity Program” (SNAP) [24] was used to assess healthy eating and physical activity behaviours of pupils over one 24-h period. SNAP is a validated, web-based programme used in years 3–6 (age 7–11 years) and designed to be a quick and easy method of assessing energy balance-related behaviours at a population level. SNAP was used in the year 4 pupils at baseline and 18 months. For the year 2 pupils, it was only used at 18 months as it is not validated for children under the age of 7 years; as a consequence, only 18 months data is presented.
- Psychological well-being of the pupils was assessed to determine whether the intervention caused any harm. It was evaluated using two validated measures:
  - The Body Shape Perception Scale (BSPS) [25]. This scale has good test-retest reliability in pupils aged 8 years or older [26] and has been regularly used in research for pupils as young as 5 years. A score of 0 indicates satisfaction with body shape; a negative value (BSPS < 0) indicates a desire to be larger, and positive values (BSPS > 0) indicate a desire to be thinner.
  - Dieting behaviours [27]: six statements from the Dutch Eating Behaviour Questionnaire (DEBQ) which comprise the dietary restraint subscale were used, as well as a further two questions from the questionnaire that refers to regulating weight by exercise and parental influences on eating behaviour.
- Pupil’s heights and weights were measured at baseline and 18 months using the procedure recommended by the NCMP guidance [28].

### Blinding

Although schools and pupils were not blinded to their allocation, the researchers collecting the outcome assessments were not informed about the intervention status of the schools.

### Statistical analysis

The study was not powered to detect changes in outcome measures; rather, the appropriateness of outcome measures for the study population were assessed by reviewing the level of missing data and assessing any floor/ceiling effects at baseline and 18 months (end of intervention). Pupil-level baseline characteristics are summarised descriptively. Due to small sample size, cluster level summaries were utilised for all outcome measures to account for clustering [29]. Cluster level averages from the HLKQ were compared between the intervention and control groups at 18 months using a linear model accounting for the minimisation factors (size of school, ethnicity, free school meal index as proxy for SES), year group, and baseline healthy lifestyle knowledge score. Estimates and 95% confidence intervals are reported for the difference between intervention and control groups.

Dietary intake data from SNAP was summarised as counts per day (number of times consumed per day). The proportion of pupils achieving a good outcome for body satisfaction (no desire to be thinner or larger) was reported as a percentage. The “dietary restraint” subscale of the Dutch Eating Behaviour Questionnaire DEBQ was scored from 0 to 12 based on pupil’s responses. They scored a zero for each “never” response, 1 for each “sometimes” response, and 2 for each “often” response, and mean dietary restraint scores are presented. Heights and weights were converted to BMI. Age and sex-specific centiles were calculated using the WHO 2006 growth reference standards [30]. BMI was converted to BMI SD scores using the LMS Growth software [31].

Effect sizes, typical cluster sizes, and 95% confidence intervals were calculated for endpoints of interest in order to inform future sample size calculations.

### Qualitative data analysis

Interviews with head teachers, healthy school co-ordinators, teachers, and focus groups with pupils were analysed using critical listening procedures and standard thematic analysis techniques [32]. Interviews and focus groups were audio-recorded and transcribed using a process of critical listening, which involves extracting and recording key information from the conversation. A coding framework using an inductive approach was developed to identify the full range of emerging themes from the data and applied to each transcript and the data organised into major thematic categories and sub-categories [32]. These themes were then discussed and agreed upon within the research team. The findings present a synthesis of the key themes in relation to the acceptability and delivery of the programme. The resources checklist was used to analyse the number and type of resources utilised by the teachers in programme delivery.

## Results

### Recruitment, randomisation, and retention

**Fig. 2:**
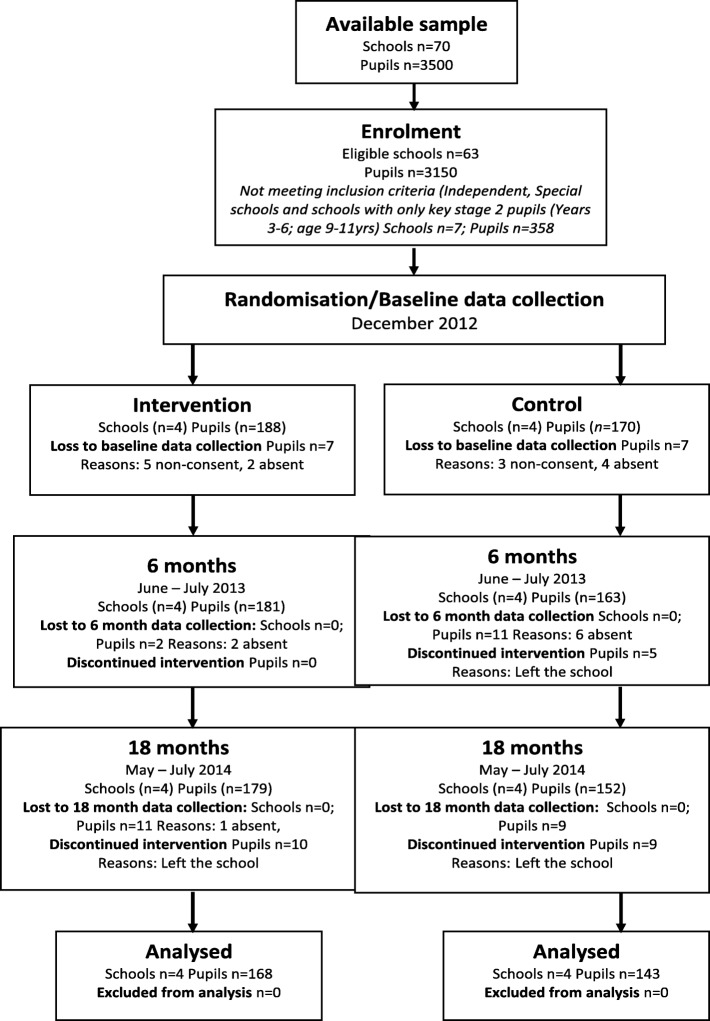
Consort flow diagram

A sample size of eight schools were recruited over a 3-month period from a town in the north of England (four intervention and four control). Individual pupil numbers fluctuated slightly over the intervention period, but only a small proportion (six pupils) opted out of consent at the start of the trial (please see Fig. 2).

The recruitment process was hindered only by timing as it commenced in July, which proved to be too late in the school calendar for schools to consider implementing a new intervention. Staff were preoccupied with end-of-term activities and curriculum planning for the following academic year. Schools were then approached from September to October 2012 and eight schools that showed interest in participating were successfully recruited. A low number of schools overall showed interest in participating due to the timing of recruitment. The teacher interviews highlighted that they required time for familiarisation with the PFP resources through additional curriculum planning time before implementing the programme.

### Cluster characteristics

School characteristics were well-balanced across intervention and control group by the minimisation factors, class size, and BME (Table 1). However there were differences in percentage of pupils eligible for free school meals between intervention and control groups suggesting free school meal eligibility a poor stratification variable. More of the control schools had greater than 17% of pupils eligible for free school meals and more children categorised as overweight/obese (Table 2).

**Table 2:** Baseline characteristics of the children by school year and intervention allocation

	Year 2		Year 4		All	
	Control	Intervention	Control	Intervention	Control	Intervention
Number of pupils	91	97	79	91	170	188
Age of pupils (years)	6.3 (0.5)	6.2 (0.4)	8.3 (0.5)	8.3 (0.5)	7.2 (1.1)	7.2 (1.1)
Male (%)	48.4	51.6	54.4	50.6	51.2	51.1
Ethnicity (%)						
White British	68.1	75.3	63.3	72.5	65.9	73.9
Pakistani	16.5	18.6	20.3	19.8	18.2	19.2
Gypsy/Roma	8.8	0.0	10.1	0.0	9.4	0.0
Weight (%)						
Underweight	0.0	0.0	0.0	3.5	0.0	1.7
Healthy weight	70.9	81.9	76.0	77.9	73.3	80.0
Overweight	10.5	6.4	10.7	5.8	10.6	6.1
Obese	18.6	11.7	13.3	12.8	16.1	12.2
BMI SDS	0.5 (1.2)	0.1 (1.1)	0.3 (1.2)	0.2 (1.1)	0.2 (1.2)	0.2 (1.1)

Data are mean and (SD) or percentages

*SDS* standard deviation scores, *Underweight* ≤ 2nd centile, *healthy weight* > 2–< 85th centile, *Overweight* ≥ 85th–≥ 95th centile, *Obese* ≥ 95th centile

*BME* Black and Minority Ethnic, *FSM* free school meal eligibility (proxy for socio-economic status)

Table 2 displays the baseline characteristics of pupils by randomised group. Of the 358 pupils registered to schools involved in the study, 51.1% were male. There was a high diversity of ethnicities within the sample, representative of the local area, thereby increasing generalisability of the findings to other ethnically diverse areas. The ethnicitities were well balanced apart from a higher percentage of gypsy/Roma children in the control schools.

### Programme delivery and acceptability

The staff interviews provided a comprehensive overview of how PFP was delivered in the intervention schools. The interviews and resources checklists that evaluated the use of the available programme lesson plans revealed substantial evidence that lesson plans had been implemented, with many intervention school teachers reporting use of some of the healthy eating lesson plans from March 2013 to July 2014. A table demonstrating how the PhunkyFoods programme was implemented at each intervention school is included as a supplementary file (Additional file 1). The PFP was designed to be flexible in delivery; therefore, the reported number and types of lesson plans delivered by teaching staff during this period varied at each school. All four intervention schools used some of the healthy eating lesson plans (three schools used more than the other). There was limited use of the physical activity lesson plans: teaching staff from only one school had used some of the physical activity lesson plans. This was because of the considerable external support received during the intervention period from specialist physical education providers into their PE curriculum. One school delivered a weekly after-school PhunkyClub during the intervention period, in addition to delivery of some of the healthy eating lesson plans, to different year groups within the classroom. The PhunkyClub was delivered to key stage 1 pupils (year 1 and year 2) and delivered interactive and practical learning on healthy eating using the online PhunkyClub curriculum (lesson plans) and “physical” resources, such as “Phunkycards” (to sort foods into food groups) and plastic food models from the resource box. A further school was in discussions around establishing a cooking club. Generally new members of staff had limited awareness of the PFP and reported only using the resources if they had been incorporated into lesson plans by the previous class teacher.

Teachers independently decided which elements of the programme they wanted to use within their lessons. Staff would generally choose the healthy eating lesson plans that addressed the topics or activities that supported the current class curriculum and were considered most engaging for the pupils, which tended to be the more practical, interactive, and “hands on” lessons. In addition, lesson plans that included practical and creative activities were selected for the after school PhunkyClub at one school, e.g. food handling, growing and tasting food, as they were considered the most engaging and favourable by pupils. Teaching staff at three of the schools also reported using “physical” resources from the resource box to enhance their healthy eating teaching, such as DVDs, food models, pictures, sorting cards (Phunkycards), books, and a food mat. The interactive resources from the resource box provided as part of the PFP were considered to make the learning experience more memorable for younger pupils. There were requests from all teachers for more interactive lesson plans with opportunities for more practical experiences for pupils.

### Resources checklists

For the teaching staff who did not complete the resources checklist at 6 months follow-up, they were provided with paper copies at 18 months follow-up in attempt to capture this data. Teaching staff at only two of the schools provided a rating of acceptability for the healthy eating lesson plans used on a likert-type scale from 1 to 5 (1 = very poor, 2 = poor, 3 = acceptable, 4 = good, 5 = excellent). The afterschool PhunkyFoods club coordinator at one of the schools also rated the rsources used. The resources that were rated were valued highly with 15 lesson plans rated as good, 9 lesson plans rated as excellent, 8 lesson plans rated as acceptable, and only 2 lesson plans rated as poor.

There were some barriers to embedding the programme fully. Initially, a small number of teachers perceived PFP as an additional activity and had expressed concerns about the limited time and appropriate lessons in which to incorporate the materials. However, following further training and a period of familiarisation with the resources and website, they were generally more receptive to the programme and able to envisage integrating it into the curriculum. Staff replacements throughout the year, although limited, meant that new staff were unfamiliar with the programme. Limited availability of staff to deliver the after school PhunkyClub throughout the year was reported by one school. Some teachers also reported a preference for other healthy lifestyle initiatives such as the Food for Life Partnership Programme (FFLP) [http://www.foodforlife.org.uk/], which staff were already familiar with and were consequently reluctant to duplicate work by incorporating an additional initiative. The Community Support Consultant role, in providing support and facilitating working in partnership with other providers to prevent duplication, was reported to be helpful. Teachers from two schools expressed concern over the inadequate facilities to deliver cooking activities suggested in some PFP lesson plans. Parents had generally not been engaged with the PFP activities, and teachers reported that parents generally resisted engaging with school activities due to time constraints and consequently perceived that involving parents was a challenge.

### Pupil focus groups

The focus groups at 18 months highlighted that there were some minor differences between intervention and control pupil’s knowledge, which was more apparent between year 2 pupils. On the whole, year 2 intervention pupils demonstrated some examples of more detailed knowledge around nutrient content of the foods and drinks displayed on the stimulus photographs, the impacts of nutrients/foods and drinks on health, and the importance of breakfast. Some year 4 intervention pupils also demonstrated more detailed and broader knowledge around nutrient content, but year 4 control pupils indicated greater sophistication of language and understanding when discussing the impacts of sugar on dental health. Control and intervention pupils in year 2 could not all accurately recall the five food groups of the Eat Well Plate [33] whereas year 4 intervention pupils were better at this than the control groups. All year 2 and year 4 pupils were able to recall some of the learning over the last year regarding healthy eating and physical activity. All four intervention schools had some awareness of the PFP, and some pupils had knowledge of specific activities related to the programme, e.g. PhunkyClub activities, a food preparation activity, and lessons.

### The Healthy Lifestyle Knowledge Questionnaire (HLKQ)

Separate age appropriate versions of the HLKQ were successfully administered to the pupils, as demonstrated by low levels of missing data (between 5 and 8% missing data across the domains) and high completion rates (96% at baseline, 94% at 6 months, and 90% at 18 months). Providing pupils with the option of writing “don’t know” was essential for multiple choice questions in order to reduce the prospect of pupils guessing the answer at baseline or follow-up, which would have made it difficult to determine what they had learnt during the intervention period.

### Attitudes to healthy foods and physical activity (year 4 only)

**Fig. 3:**
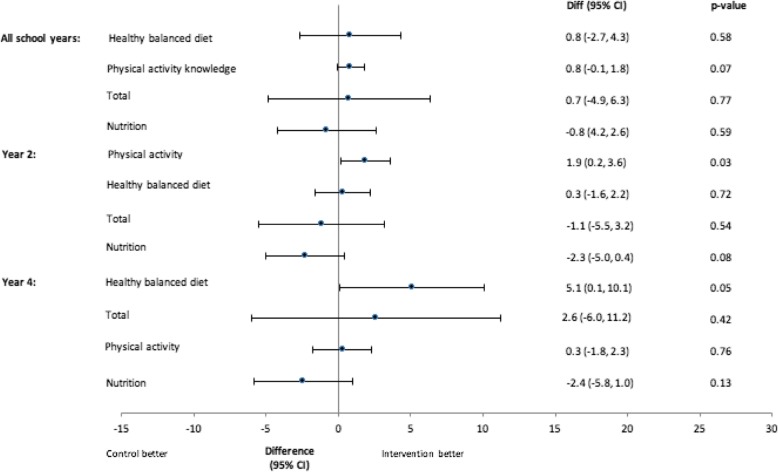
Mean difference in Healthy Lifestyle Knowledge Questionnaire scores (18 months)

Figure 3 shows the results from the linear model comparing HLKQ scores at 18 months. Confidence intervals are wide reflecting the small sample size, and there are no differences between groups for the year 2 and year 4 pupils combined. There was a trend towards higher physical activity knowledge scores in the intervention group compared to the control group, mean difference 0.8 (95% confidence interval − 0.1 to 1.8), *p* = 0.07. When considering the year groups separately, the year 2 intervention pupils had significantly higher physical activity knowledge scores at 18 months compared to the year 2 control pupils, mean difference 1.9 (95% confidence interval 0.2 to 3.6), *p* = 0.03. Year 4 intervention pupils had significantly higher healthy balanced diet scores compared to year 4 control pupils, mean difference 5.1 (95% confidence interval 0.1 to 10.1), *p* = 0.05. There was a trend towards higher nutrition scores for the control group in year 2, mean difference − 2.3 (95% confidence interval − 5.0 to 0.4), *p* = 0.08.

The year 4 pupils completed a questionnaire exploring their attitudes towards fruit and vegetables and physical activity. The pupils were asked to tick if they had never had it or tried the fruit/vegetable/activity, Yes, they liked it or No I don’t like it. The percentage of vegetables, fruits, and sports liked increased from baseline to 18 months for all pupils, as shown in Table 3. At 18 months, the mean percentage of vegetables liked was higher across the intervention group compared to the control group (53.9% vs. 43.0%). Similarly, at 18 months, the percentage of fruits liked was also higher in the intervention group (76.9% vs. 67.2%). The percentage of sports liked was similar between the two groups at 18 months.

**Table 3:** Pupil’s preferences towards vegetables, fruits, and sports by intervention allocation

	Baseline		6 Months		18 months	
	Control	Intervention	Control	Intervention	Control	Intervention
Liked vegetables	42.8 (19.9)	43.1 (19.1)	43.6 (21.3)	47.6 (20.1)	43.0 (17.9)	53.9 (19.6)
Liked fruit	62.5 (25.0)	66.7 (23.0)	69.5 (26.4)	74.6 (25.4)	67.2 (24.7)	76.9 (21.9)
Sport liked	53.3 (27.2)	58.9 (25.1)	62.8 (25.4)	60.0 (25.3)	67.7 (19.0)	67.9 (21.5)
*N*	76	84	68	83	71	84

% liked (SD)

### Synchronised Nutrition and Activity Program (SNAP)

The level of missing data was low (only 6% at baseline and 10% at 18 months). Table 4 shows the frequency of consumption per day (number of times food or drink item consumed per day) at 18 months. Baseline data for this analysis was only available for year 4 children, as the tool was not validated in the younger year group. Mean intake levels were low due to a high proportion of pupils recording zero counts. At 18 months, the reported intake of full sugar fizzy drinks, chocolate/biscuits, sweets, cakes, ice cream, custard, crisps, and takeaway style foods (pizza, kebabs, burgers, Chinese, curry, battered sausages, and fish) was slightly lower in the intervention group compared to the control group and reported intake of vegetables and pies and pasties slightly higher.

**Table 4:** Frequency of food and drink consumption per day at 18 months by intervention allocation

	Year 2		Year 4		All	
	Control	Interv	Control	Interv	Control	Interv
Water	1.8 (1.5)	1.7 (1.3)	1.6 (1.3)	1.8 (1.5)	1.8 (0.2)	1.7 (0.4)
Diet fizzy drinks	0.1 (0.4)	0.1 (0.4)	0.1 (0.3)	0.1 (0.2)	0.1 (0.1)	0.1 (0.1)
Full-sugar fizzy drinks	1.6 (0.9)	1.3 (0.5)	1.6 (1.0)	1.3 (1.7)	0.3 (0.2)	0.2 (0.1)
Chocolate, biscuits, sweets, cakes, ice cream, custard	2.1 (1.7)	1.8 (1.3)	2.7 (1.7)	2.1 (1.5)	1.8 (0.3)	1.7 (0.5)
Pies and pasties	1 (0.0)	1.0 (0.0)	1 (0.0)	1.0 (0.4)	0.0 (0.0)	0.2 (0.2)
Chips	1.2 (0.4)	1.1 (0.3)	1.2 (0.4)	1.1 (0.3)	0.5 (0.2)	0.5 (0.2)
Takeaway-style foods (pizza, kebabs, burgers, Chinese, curry, battered sausages, and fish)	1.2 (0.5)	1.2 (0.5)	1.6 (0.8)	1.4 (0.5)	0.7 (0.1)	0.4 (0.2)
Crisps	1.4 (0.6)	1.3 (0.5)	1.2 (0.4)	1.4 (0.7)	0.4 (0.2)	0.3 (0.1)
Meat and meat alternatives (including sausages—not takeaway)	1.4 (0.7)	1.5 (0.7)	1.6 (0.8)	1.4 (0.6)	0.7 (0.4)	0.9 (0.2)
Fruit (including dried fruit)	1.4 (0.8)	1.4 (0.9)	1.5 (1.0)	1.6 (0.9)	0.6 (0.1)	0.6 (0.3)
Vegetables (including tomatoes, beans, pulses)	1.3 (0.6)	1.4 (0.6)	1.2 (0.5)	1.4 (0.6)	0.7 (0.2)	0.8 (0.2)

Data are mean and (SD)

### Psychological well-being

The proportion of missing data for the dietary restraint questionnaire and body shape satisfaction questionnaire was 11% and 12% respectively. As the questionnaires address what are generally perceived to be more “sensitive issues” such as body awareness and dieting behaviours, this may account for the greater proportion of missing data. A standardised script explaining there was no correct or incorrect answer and assuring confidentiality of responses was used to offer reassurance with completion. However, the larger class size may have hindered completion: therefore, smaller group sizes and to offer assistance as required may improve completion rates.

### Body shape satisfaction

For the proportion of pupils reporting body shape satisfaction at 18 months, where satisfaction was defined as remaining at zero or reaching zero (as zero represents no desire to be thinner or bigger), at baseline, there was very little difference between the intervention and control groups with 33.3% (*N* = 167) of the control group and 38.5% (*N* = 148) of the intervention group reporting body shape satisfaction. At 6 months, there was a higher proportion of the intervention group reporting body shape satisfaction, but by 18 months, the percentage was similar, 50.6% (*n* = 158) in the control group versus 41.4% (*n* = 128) in the intervention group. The results suggest that there was no negative impact on body image to pupils through taking part in the PFP intervention.

### Dieting behaviour

Table 5 summarises the average total scores for items on the dietary restraint questionnaire across year group and intervention group. Dietary restraint scores range from 0 to 12 with 0 reflecting low dietary restraint. Overall scores reflected low to moderate levels of dietary restraint in this sample. Scores were similar in the intervention and control groups. There were higher levels of missing data in the control group overall as one control school refused consent for their pupils to complete the dietary restraint questionnaire.

**Table 5:** Psychological well-being: dietary restraint

		Year 2						Year 4					
		Baseline		6 Months		18 months		Baseline		6 Months		18 months	
		Cont.	Int.	Cont.	Int.	Cont.	Int.	Cont.	Int.	Cont.	Int.	Cont.	Int.
Dietary restraint	*N*	81	88	78	85	75	85	67	83	58	79	63	88
	Mean (SD)	3.2 (2.8)	4.2 (3.0)	5.0 (2.8)	4.8 (2.5)	6.5 (2.9)	5.9 (2.7)	5.5 (2.5)	5.3 (2.9)	5.6 (2.6)	5.6 (2.6)	5.1 (2.9)	5.3 (3.0)
	*N* missing	10	9	13	12	17	13	12	8	21	12	16	7

Dietary restraint scores range 0–12 with low score representing low dietary restraint

*Cont.* control, *Int.* intervention

### BMI SD scores

Over the 18-month intervention period, BMI SD scores in the control group had a mean change of − 0.07 (95% CI − 0.56, 0.20) and the intervention group a mean change of 0.12 (95% CI − 0.2, 0.45).

## Discussion

The Medical Research Council Framework for Developing and Evaluating Complex Interventions outlines the importance of conducting feasibility and pilot studies to examine complex interventions such as the PFP prior to undertaking a phase 3 trial [34]. The findings of this study have demonstrated the feasibility of delivering the PFP which targets dietary and physical activity knowledge and behaviours in primary school children and its acceptability to teachers and pupils. The study has generated information to enhance the programme content and delivery and also provided information on the appropriateness of the outcome measures to inform the definitive cluster randomised trial. Although the feasibility study was not powered to stastistically examine intervention outcomes, there were trends in increased knowledge of healthy lifestyle behaviours, healthier eating, and liking of fruit and vegetables reported in the intervention group compared to the control group. The findings suggest that a full phase 3 trial is feasible, with some minor modification to the study design tools used to evaluate the intervention.

### Recruitment, sample size, and adherence

For a phase 3 trial, the recommendation is to recruit earlier in the year, preferably in January/February with baseline measurements taken in March/April and then for the schools to be randomised. This would then provide a time during May–July for teacher training and to offer the necessary familiarisation and planning time to implement the intervention in September, at the start of the academic year.

### Sample size for full trial

The nature of the intervention and observed trends from this feasibility study suggest that improvement in dietary behaviours such as intake of fruit and vegetables may be a good primary outcome for future research as fruit and/or vegetables are considered key indicators of change in school-based intervention programs [35]. To calculate the sample size for a fully powered trial based on fruit and vegetable intake as the primary outcome, we propose using the average portions of fruit and vegetables consumed per day across the sample at baseline which was 1.3 (SD 1.3). On the assumption of attempting to increase pupil’s intake by half a portion, a future phase 3 trial would need 216 pupils (based on 80% power and 5% level of significance) [36]. The size of the effect of the study is powered to detect one half of a portion of vegetables or one portion of fruit and was chosen because it was considered the smallest improvement in intake that was worthwhile detecting with the achievable sample size, considering the nature of the intervention [37]. The interclass correlation coefficent from the feasibility study was 0.07; therefore, the sample size would need to be inflated by a factor of 5.13 assuming a cluster size of 60. The total sample size required for the cluster trial would therefore be 1108 pupils. This would mean that a future trial would involve 22 clusters (11 schools per intervention group) to allow for up to 20% pupil dropout.

### Assessement tools

The total knowledge score, healthy balanced diet knowledge score, and nutrition knowledge score from the HLKQ did not show any difference between the intervention groups. Despite the small sample size, there was a trend towards higher physical activity knowledge scores in the intervention group compared with the control group (mean difference 1.9). This effect was statistically significant for the year 2 pupils at 18 month. It is unlikely that this effect is due to the intervention, because there was limited use of the PhunkyFoods physical activity resources within each school curriculum. Furthermore, the year 4 pupils did not show the same trend in physical activity. For year 4, there was a statistically significant, higher mean healthy balanced diet knowledge score in the intervention group versus the control (mean difference 5.1). However, this was not demonstrated in the year 2 pupils. These results support previous research that nutrition knowledge has been shown to be positively related to improved dietary habits [38]. Whilst this study was not powered to show a significant difference, it is evident that there might be a trend that the intervention is affecting the nutrition knowledge of pupils.

The SNAP diet and physical activity assessment tool identified indications of some small differences between the intervention and the control groups. Although not powered to examine intervention outcomes, the direction of effect for dietary outcomes (i.e. foods hypothesised to be related to obesity development) was in favour of the intervention. However, no meaningful trend was observed between the intervention and control group pupils for MVPA or in pupils meeting the recommended 60 min of MVPA per day. SNAP is validated for use in the year 4 pupils, due to the web interface requiring no data entry. However, there were implementation issues: although validated in year 4 pupils, they still required assistance to complete it accurately; it worked slowly on old computers; and not all the data was recorded at baseline and 6 months (e.g. minutes of MVPA were not recorded despite the data being inputted). Consequently, the results for the physical activity data show the amount of total physical activity undertaken but not MVPA at baseline and 6 months. For a future trial, the best method to measure physical activity would be to use pedometers for 1 week during waking hours for the whole sample and in a sub-sample to use ActiGraphc acceleormeters that would provide a detailed analysis of movement and capture any differences between the intervention and the control group [2, 39]. This methodology has been sucesssfully used in several school-based intervention studies [39–41]. For dietary analysis, a tool that is validated for both year groups such as the Child Dietary and Assessment Tool (CADET) [42], a 24-h dietary recall assessment tool, or the Children’s Dietary Questionnaire (CDQ), which is a 28-item semi-quantitative food-frequency questionnaire [43], should be used.

The results from the psychological wellbeing scales suggested that no psychological detriment had occurred as a result of pupils participating in the intervention group. Therefore, for a future phase 3 trial, it is suggested to conduct focus groups in a subsample to explore pupils’, teachers’, and parents’ experiences and perceptions of the intervention. This would reduce time spent gathering data in the classroom for the study, whilst still collecting vital process measures information. A similar methodology has been used in other school-based interventions [39].

Although over the 18-month intervention period more of the intervention group pupils compared to the control group pupils moved from a healthy weight to overweight and obese categories, the trend is too small to be indicative of any intervention effect. However, this trend is noted and highly likely mirrors the rapid rise in overweight and obesity levels between the ages of 4–5 years and 10–11 years as reflected in data from the National Child Measurement Programme [44], and it will have to be closely monitored in the phase 3 trial.

## Programme delivery

All four intervention schools delivered some of the PFP lesson plans (three schools had used more of the lesson plans than the other school). More of the PhunkyFoods healthy eating curriculum (lesson plans) was delivered than the physical activity curriculum. The PFP was designed to be flexible in delivery so that schools could choose which elements of the programme they wanted to deliver as part of the school curriculum. Therefore, the types and number of lesson plans used varied at each school and only one school provided an additional after school PhunkyClub. As schools were in the early stages of implementation and “trialling” lesson plans, they were mainly selecting those that were easily incorporated within their current curriculum topics and those that they felt were more engaging for pupils, which tended to be the more interactive and practical lesson plans. A previous thematic synthesis of process evaluation data from 26 studies included in a Cochrane review of WHO’s Health Promoting Schools Framework (including two studies conducted in primary schools in the UK) [45] highlighted that schools desire interventions to be flexible and tailored to the local contexts of the school. The flexibility of the PFP, with classroom delivery or afterschool club delivery and flexible delivery of lesson plans and activities, is therefore a strong programme attribute. Providing teachers with a resources checklist that listed all the PFP curriculum (lesson plans) proved a useful strategy for assessing which lesson plans for teaching had been used; however, they were not all completed fully. Although in the interviews teachers reported high acceptability of the programme, in order to evaluate implementation of the programme more robustly including frequency of use of the resources, it is recommended that in the phase 3 trial, the resources checklists need to be completed concurrently with programme implementation, by all teaching staff involved with programme delivery.

The quality of resources and activities was rated highly by the teachers and pupils in the focus groups. However, in order to strengthen programme delivery, teacher training should to be delivered in May/July, allowing sufficient time for familiarisation and curriculum planning. The training should also be more practical and include activities such as lesson planning and familiarisation with the website/resources with follow-up support from the Community Support Consultant. Staff replacements throughout the year meant that new members of staff were unfamiliar with the programme, and therefore, it is recommended that additional training would be helpful for new staff. Community Support Consultant input was valued and therefore should be maintained.

### The PhunkyFoods intervention

The PFP programme supports the current policy drivers aimed at addressing childhood obesity [46, 47] by targeting risk factors associated with obesity development and is well-placed to play a pivotal role in supporting the school food agenda aimed at addressing inequalities and the learning gaps. A recent systematic review highlighted how important public health interventions are at creating small positive significant changes to long-term health [48]. The strengths of PFP lie in its multicomponent nature that targets both diet and physical activity and thereby supports the evidence from recent systematic reviews [14, 49] and reports [16, 46, 47] that suggest multi-component school-based approaches have potential to promote healthy lifestyle behaviours. Current thinking about health education in schools envisages a whole school approach and encompasses the taught curriculum: the hidden curriculum such as school food policies, e.g. breaktime snack policies, lunchtime provision, and a supportive physical environment such as pleasant dining room facilities and playgrounds and the active participation of parents and governors, teachers and pupils, and the wider community including other agencies. The pedagogic approach adopts a spiral curriculum where health education is provided for all ages in a cross-curricular fashion and one that is child-centred. The aim is to offer a positive healthy lifestyle approach across the whole school with extending links into home.

The delivery of PFP fits well into the whole school approach where the range of lesson plans support the curriculum in the area of healthy eating and physical activity across curriculum topic areas. The range of PFP lesson plans offer the potential to embed the programme within the curriculum throughout primary school so that the topic is not viewed by pupils or teachers as a one-off activity but revisited at appropriate time points throughout primary school. This is key to the promotion of sustained knowledge and healthy behaviours. Pupils recalled some of the PFP activities related to the programme and rated them as fun and enjoyable. Additionally, some activities were recalled from the previous year which is encouraging. School-based interventions such as PFP work well in schools as they are not a standarised fixed intervention. Teachers are able to select from a range of activities and lesson plans and tailor intervention programmes to their individual school needs [48]. A key component of the overall structural design of the PFP is that the breakfast and after school clubs provide flexibility to deliver the programme outside the formal curriculum whilst at the same time supporting the curriculum. The role of Community Support Consultants also provides the important support for staff within school and between school, home, and the wider community.

However, a recommendation for strengthening the PFP would be to consider options to actively involve parents due to their important role in supporting and maintaining healthy behaviours outside the school environment. There were also requests from all teachers for more interactive lesson plans with opportunities for more practical experiences for pupils, as these were well received by pupils. A recent systematic review has reported that experiential learning strategies were associated with the largest effects in reducing food consumption or energy intake, increased fruit and vegetable consumption or preference, and increasing nutritional knowledge outcomes]. Reducing sugar consumption and preference was most influenced by cross-curricular approaches embedded in the interventions [14].

There was limited reported use of physical activity resources, and teachers reported that this was due to the high level of support available for the physical education curriculum at the time of the study. Therefore, at recruitment, the future study will need to ascertain whether schools are enagaged or have access to additional healthy lifestyle programmes which may influence programme fidelity and outcomes, as the literature has found physical activity to be a fundamental component of effective obesity prevention programmes [15, 50].

The current literature still advocates that we are unsure of the required dose of a behavioural intervention necessary to prevent childhood obesity. Conducting a full randomised control trial on programmes such as the PFP is vital to improve our understanding of effective preventative programmes [50].

### Strengths and limitations

Recruitment, retention, and response rates were high and the study has provided important information on acceptability and feasibility of the intervention. One of the key reasons for a high response rate for data collection was due to two separate opportunities built into the design for data collection. Nevertheless, over a 2-year period, there was still missing data. It was higher amongst the control group pupils for SNAP, psychological measures, and heights and weights, whereas the level of missing data from the HLKQ was higher for the intervention group pupils. The processes for data collection were identical in the intervention and control schools, and this will need to be monitored closely and strategies employed to minimise the level of missing data in the phase 3 trial, e.g. data collection in smaller groups instead of as a whole class. Delivery of the PhunkyFoods intervention was non-standardised and undertaken by staff outside the research team. This was considered a strength as it allowed a pragmatic approach to be tested, which could be more easily rolled out.

A limitation was the SNAP programme, as it was not validated to be used in children under 7 years of age. Future phase 3 trial should identify a validated tool such as Child and Diet Evaluation Tool [42] or the Children’s Dietary Questionnaire (CDQ) [51],validated for collecting dietary recall information from children from the age of 6 years would be used in the full trial. Another limitation is that there were slightly more low SES and gypsy/Roma children in the control group compared to the intervention group. Despite using randomisation to reduce these occurrences, the imbalance can potentially affect the primary outcome results, with such a small sample. Although this should mitigated in a larger sample, baseline imbalances in SES and ethnicity due to potential impact on the primary outcome should be considered in the main trial. The trial was conducted within a single site, which may not be generalisable to other locations; however, the sample of schools included a representative, ethnically diverse population, and the intervention was delivered independently from the research team. A limitation is that the costs of the intervention were not formally examined as this was a feasibility trial to assess acceptability of the programme.

## Conclusion

The study has provided important information on acceptability and feasibility of the PFP and recommendations to enhance the programme and its delivery. It has also provided evidence on the appropriateness of the outcome measures and sample size to inform the definitive cluster randomised trial. These early findings and lessons learned suggest that that a full trial to evaluate the effectiveness and cost-effectiveness of the PFP is feasible, with some minor modification to the study design and assessment tools.

## Supplementary information

**Additional file 1.** The delivery of the PhunkyFoods programme within each Intervention school: mode of delivery and types of resources used. Rating of online resources from 1 to 5 (1 = very poor, 2 = poor, 3 = acceptable, 4 = good, 5 = very good) were provided by teaching staff

## Data Availability

The study dataset is available on request from the study investigators.

## Abbreviations

PFP: PhunkyFoods Programme
UK: United Kingdom
SES: Social economic status
NCMP: UK National Child Measurement
FSM: Free school meal
FFLP: Food for Life Partnership Programme
BME: Black and Minority Ethnic
BMI SD: Body mass index standard deviation
HLKQ: Healthy Lifestyle Knowledge Questionnaire
CADET: Child Dietary and Assessment Tool
CDQ: Children’s Dietary Questionnaire
SNAP: Synchronised Nutrition and Activity Program
MVPA: Moderate to vigorous physical activity
NICE: National Institute for Clinical Excellence
