# Supplementary material for: The feasibility and acceptability of a primary school-based programme targeting diet and physical activity: the PhunkyFoods Programme
Source: Pilot Feasibility Stud. 2019 Dec 20;5:152. doi: 10.1186/s40814-019-0542-2 (PMC6925414; doi:10.1186/s40814-019-0542-2)
Supplement: Supplementary file 1 — Additional file 1. The delivery of the PhunkyFoods programme within each Intervention school: mode of delivery and types of resources used. Rating of online resources from 1 to 5 (1 = very poor, 2 = poor, 3 = acceptable, 4 = good, 5 = very good) were provided by teaching staff [file 40814_2019_542_MOESM1_ESM.docx]

**Supplementary Table. The delivery of the PhunkyFoods programme within each Intervention school: mode of delivery and types of resources used. Rating of online resources from 1-5 (1=very poor, 2=poor, 3=acceptable, 4=good, 5=very good) where provided by teaching staff.**

| **School** | **Year groups delivered to** | **PhunkyFoods resources used** | **Rating**  **(1-5)** |
| --- | --- | --- | --- |
| School 1 | Year 5 | Lesson plans followed themes of Term 2 Diet and Health  Healthy eating quiz, energy, foods that give us energy, malnutrition, introduction to physical activity, physical activity and our body, physical activity and our heart, get active promotion, fantastically fit challenge |  |
|  | Year 3 | Current teacher had not used any plans (started in January)  Previous teacher had used lesson plans within science (healthy eating topic) - Where food comes from as part of a “healthy living day” with all year groups and watched DVDs related to this  Used ‘hard resources’ including food models, food mats |  |
|  | Year 4 | Used lesson plans relating to - foods from around the world, where food comes from; what do we need for the body, healthy diet; e.g. the world in my shopping bag, St Lucia, food shopping in Kenya, bonkers about bananas, tudor times. |  |
|  | Year 6 | Some lesson plans used during a healthy lifestyle unit – food through the ages. War time rationing (diets), history of food in history |  |
|  | Year 2 | No resources had been used by the teacher interviewed– the other part time year 2 teacher had used some within PSHE lessons and Science lessons (no examples could be given). |  |
|  | Year 1 | Used ‘hard resources’ (box of resources) 7 times (Design Technology twice, PSHE once, geography 4 times) with key stage 1 and in science - food models, pictures, sorting cards, books, floor mat |  |
|  | PhunkyFoods after school club  Key Stage One | Used the scheme of lesson plans provided  Plate of Health, Festival of Food  Living and Non-living things  Plant or animal  Harvest festival  Granddad the Greengrocer  Growing Cress  Facts about Fish  Fish Tasting- magic boxes  What is health?  Healthy activities  Let’s get active  Eat more of? Eat less of?  Fun with Food | 2  2  3  3  3  3  5  3  3  4  4  5 |
| School 2 | Year 1 and 2 | Used lesson plans and ‘hard resources’ (box of resources) in Science – growing vegetables (carrots, lettuce, green beans, radishes and sandwich preparation).  Also delivered in PSHE lessons, religious education and geography lessons e.g. Food form around the world.  Used flashcards and games on the healthy food plate out of the box of hard resources.  Examples of lesson plans used:  Growing potatoes  Granddad the Greengrocer  Indoor Gardens  Where can I buy food from  The Five Food groups  Food in celebration  Planning a festival (1)  A celebration of food | 4  4  4  4  3  4  4 |
|  | Year 3 | Used lesson plans:  Bread Factory  Our Food from Around the World  (used as part of wider topics on Commonwealth and Bread) |  |
|  | Year 5 and 6 | Used lesson plans:  Making compost  Love Food, Hate Waste  Left Over lunches | 4  3  4 |
| School 3 | Year 4 | Used 4 lesson plans relating to Diet and Health; history of food e.g. Egyptians and Romans and during PSHE (6 week unit on healthy lifestyle) |  |
|  | Year 3 | Within DT lessons – sandwich making |  |
|  | Year 5 | Fishing and sustainability e.g. Fish Business |  |
|  | Year 2 | Resources used within group circle work e.g. Plate of Health |  |
| School 4 | Key stage 1 and 2 | Box of resources - DVDs, flashcards and stickers, lists of foods and mix and match activities for the lower school and the power point presentations around health and hygiene were used with the older children.  Back to Basics week - leant about table etiquette, manners, food preparation activities, food tasting e.g. Ready Steady Cook activity (all year groups) |  |
|  | Year 3 | Lesson plans used in 3 topics: food glorious food, how do plants grow and food from other countries. Also used growing resources and lesson plans in relation to dental health  Other lesson plans used:  It’s all about balance  Food to make our body work  We all need food from somewhere  The Plate of Health Recap  Healthy Choices | 4  4  4  4  4 |
|  | Year 2 | Used lesson plans:  The five food groups  Eat more of…eat less of (1)  Eat more of…eat less of (2)  Strive for 5  Eat a Rainbow  Fruit Salad  Do we have a balanced diet?  My balanced breakfast | 5  5  5  5  5  5  5  5 |
|  | Year 4 | Lesson plans for Stone Age, Romans, Tudors, Food hygiene |  |
|  | Year 5 | No resources used (new teacher) |  |
